# Advances in the management of craniopharyngioma

**DOI:** 10.12688/f1000research.15834.1

**Published:** 2018-10-11

**Authors:** Lillie O'steen, Daniel J. Indelicato

**Affiliations:** 1 Department of Radiation Oncology, University of Florida, 2000 SW Archer Road, Gainesville, FL 32610, USA; 2 Department of Radiation Oncology, University of Florida, 2015 North Jefferson Street, Jacksonville, FL 32206, USA

**Keywords:** Benign, pediatric, radiation therapy, surgery, outcomes, treatment side effects

## Abstract

Craniopharyngioma is a curable benign tumor, but owing to its intimate relationship to critical structures in the central brain—such as the optic apparatus, pituitary, hypothalamus, intracranial vasculature, brain stem, and temporal lobes—its management introduces the risk of long-term treatment morbidity. Today, the most common treatment approach is conservative subtotal resection followed by radiotherapy, and the goal is to limit long-term toxicity. Many recent advances in the treatment of craniopharyngioma are attributable to improved surgical techniques and radiotherapy technologies.

## Introduction

Craniopharyngioma is a benign tumor typically treated with both surgery and radiation, an approach that offers 5-year progression-free survival (PFS) rates exceeding 90% ^1^. Historically, these high tumor control rates have come at the cost of long-term side effects, such as endocrinopathy, hypothalamic dysfunction, visual field deficits, cerebrovascular sequelae, secondary malignancies, and neurocognitive decline, which significantly impact quality of life among this mostly pediatric population.

Whereas most benign tumors can be treated surgically, craniopharyngiomas present a surgical challenge because of their central location and close proximity to sensitive structures, such as the optic apparatus, pituitary, hypothalamus, circle of Willis, brain stem, and temporal lobes. Schoenfeld *et al*. retrospectively reviewed 122 patients whose craniopharyngioma was treated between 1980 and 2009 with gross total resection (GTR) or subtotal resection (STR) and radiotherapy ^2^. GTR was associated with a significantly higher incidence of diabetes insipidus (56.3% versus 13.3%, *p* <0.001) and panhypopituitarism (54.8% versus 26.7%, *p* = 0.014) and showed no improvement in PFS or overall survival ^2^. In an analysis of 644 patients from the Surveillance, Epidemiology and End Results Program whose craniopharyngioma was treated between 2004 and 2008, Zacharia *et al*. examined factors such as younger age, smaller tumor size, and combined-modality therapy ^3^. They found that STR and radiotherapy significantly improved survival. In addition, the 10-year local control rate was higher with STR plus radiation than with surgery alone (84% versus 52%; *p* = 0.006) ^3^. STR alone has been associated with significantly inferior PFS compared with surgery and radiation ^2^. Because aggressive surgery carries a higher risk of morbidity and the rates of progression with STR alone are unsatisfactory, the standard of care for most craniopharyngiomas involves conservative surgery with the goal of preserving vision and controlling hydrocephalus, followed by radiotherapy to optimize local control. Conservative surgical resection is of particular importance in cases with radiographic evidence of hypothalamic involvement, which is associated with decreased 10-year overall survival and an enduring impact on psychosocial quality of life ^4, 5^. Only a select subset of tumors, usually small and separate from the hypothalamus and optic pathway, may be cured with surgery alone.

The most recent advances in the treatment of craniopharyngioma have focused on minimizing treatment-related toxicity. These advances include endoscopic surgery and precision radiotherapy. Radiation therapy technology has improved dose conformality and provided decreased doses to adjacent critical structures with the goal of reducing long-term sequelae in this highly curable pediatric population.

## Endoscopic endonasal surgery

Prior to the advent of endoscopy, only intrasellar, infradiaphragmatic lesions could be resected through an endonasal approach. With the advent of endoscopic endonasal surgery (EES), suprasellar and select intraventricular tumors, which were accessible only using craniotomy, can now be resected using EES, often with improved clinical outcomes compared with transcranial resection. Karavitaki *et al*. reviewed 64 craniopharyngioma patients who underwent EES ^6^. The GTR and near total resection rates were 37.5% and 34.4%, respectively, similar to historical rates with transcranial resection ^6^. There was no difference in extent of resection between intrasellar and suprasellar tumors. The rates of visual deterioration (0%) and new endocrinopathies (58.3%) were lower with EES compared with published results with transcranial resection ^7– 9^.

## Intensity-modulated photon radiation therapy

The fundamental objective of radiotherapy is to deliver a therapeutic dose to the tumor target while limiting the dose to nearby normal structures. Intensity-modulated photon radiotherapy (IMRT) is a precise radiotherapy modality that tailors small beamlets of varying intensities to a complex target structure. Compared with three-dimensional conformal radiotherapy (3DCRT), IMRT offers improved dose conformality and reduced dose to adjacent normal structures. In a dosimetric study of 15 pediatric craniopharyngioma patients who underwent treatment planning for both 3DCRT and IMRT, IMRT reduced the mean dose to the cochlea from 18.2 to 13.3 Gy ( *p* <0.001), temporal lobes from 14.3 to 7.9 Gy ( *p* <0.001), and hippocampus from 26.8 to 17.6 Gy ( *p* <0.001) ^10^.

## Proton therapy

**Figure 1.**
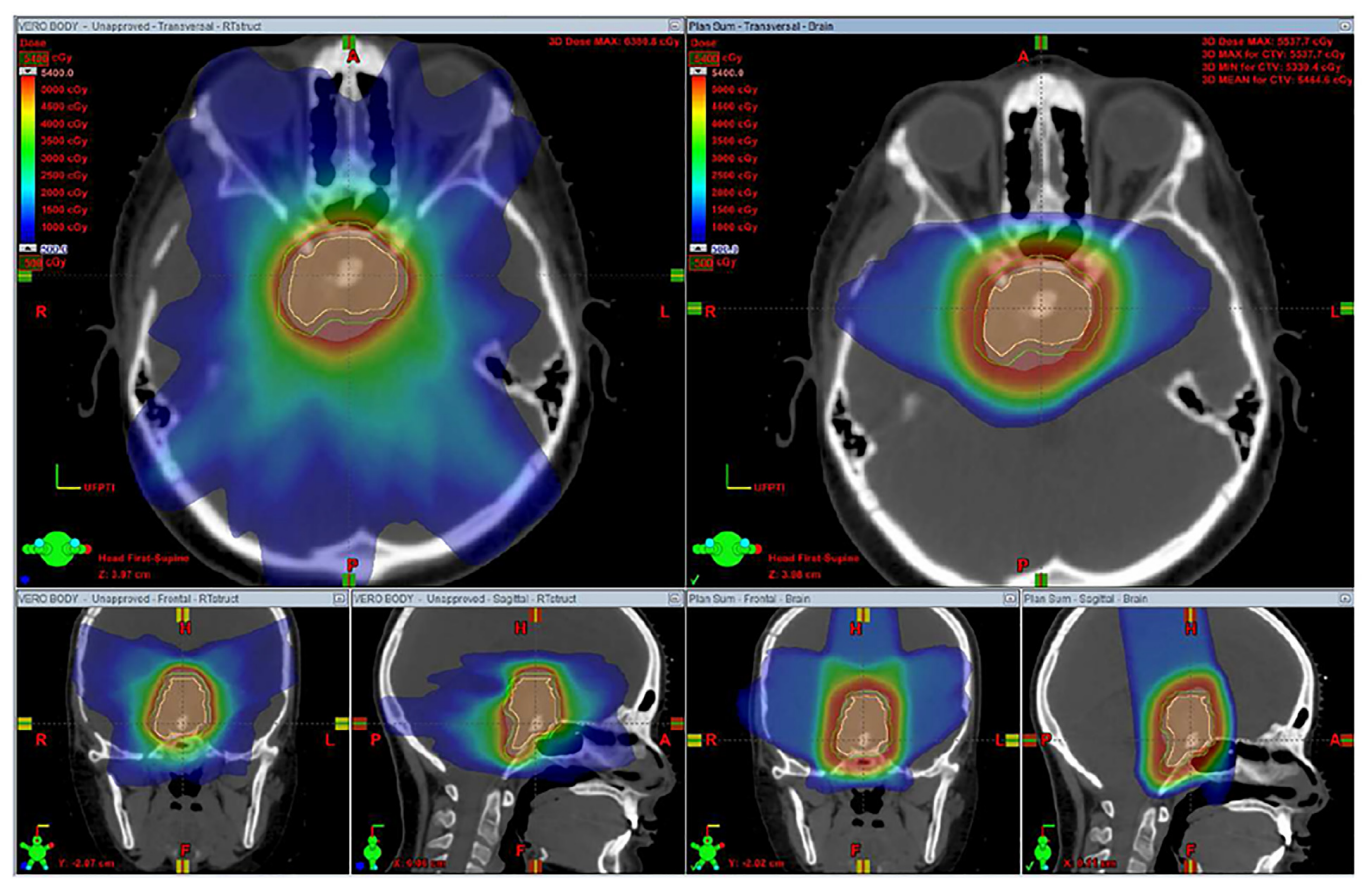
Dosimetric comparison of stereotactic radiotherapy with three-dimensional conformal proton therapy. Stereotactic radiotherapy (left) offers a sharp dose fall-off, but owing to the seven-beam arrangement with both entry and exit dose, a low dose is deposited diffusely in the region of the normal brain surrounding the central tumor. Three-dimensional conformal proton therapy (right) offers a sharp dose fall-off and, with a three-beam arrangement with no exit dose, offers less dose deposition in the normal brain tissue. The proton plan offers better sparing of the supratentorial brain and orbits. The figures are original images taken in our clinic for this publication.

Protons from a cyclotron or synchrotron travel through tissue delivering small dose until reaching their maximum depth, where, depending on their energy, they deposit a narrow distribution of dose before stopping, producing the characteristic Bragg peak. Unlike in photon-based radiation, no “exit” dose is delivered beyond the target with proton therapy. A “spread-out” Bragg peak can be created by delivering protons across a range of energies. In a dosimetric study of 10 pediatric craniopharyngioma patients who underwent treatment planning using IMRT, three-dimensional conformal proton radiotherapy (3DCPT), and intensity-modulated proton therapy (IMPT), both 3DCPT and IMPT demonstrated a relative reduction in the integral dose to the brain stem, hippocampus, dentate gyrus, vascular structures, subventricular zone, infratentorial region, supratentorial region, and whole brain ^11^. Such a dose reduction to these intimately located critical structures can help lessen the acute and late toxicities of radiotherapy. Investigators of a Childhood Cancer Survivor Study analyzing pediatric patients with a variety of tumors calculated a 2- to 15-fold reduction in the incidence of second malignancies when proton therapy replaces conventional photon radiotherapy ^12^. Compared with photon therapy, proton therapy offers a better opportunity to preserve IQ scores in patients with craniopharyngioma ^13^. In a review of 40 pediatric craniopharyngioma patients who received proton radiotherapy, the 5-year local control and overall survival rates were 100% ^14^. Table 1 reviews the published outcomes on patients with craniopharyngioma treated with proton therapy ^14– 22^. A comparison of photon stereotactic radiotherapy and 3DCPT plans is shown in Figure 1.

**Table 1.** Outcomes following proton therapy for patients with craniopharyngioma.

Study; number of patients	Median follow- up, years	Treatment modality	Actuarial 5-year local control rate	Acute toxicity, number of patients	Late toxicity, number of patients	Mortality (absolute number), number of patients
Fitzek *et al*. ^15^ (2006); N = 15	13.1	Surgery/biopsy + proton-photon	93% 5-year	None, 7; nausea, 1; fatigue, 3; headaches, 4	Visual deficits, 2; endocrinopathy, 15; learning difficulty, 1	PD, 2; vascular complications, 1; treatment related hypothalamic syndrome, 1
Luu *et al*. ^16^ (2006); N = 16	5	Surgery + proton or proton alone (surgery + RT, 4; recurrent after surgery, 12; re-resection + RT, 7; RT, 4)	94% ^a^ (recurrence at 80 months, 1)	NR	Panhypopituitarism, 1; CVA, 1 with full recovery; meningioma, 1 following re-irradiation	3 at 12, 52, and 120 months after re-resection and RT (PD, 1; sepsis, 1; MCA infarct, 1)
Winkfield *et al*. ^17^ (2008); N = 24	3.7	Surgery/biopsy + proton therapy	100%	NR	NR	Intracranial hemorrhage at 1 year, 1 (in a child with 3 previous surgeries followed by RT)
Chang *et al*. ^18^ (2009); N = 14	1.3	Surgery/biopsy + proton therapy	100% ^a^	NR	Vision, stable or improved; endocrinopathy, 11 (of 11 with results)	0
Alapetite *et al*. ^19^ (2012); N = 49	4.4	Surgery + proton- photon, 10; surgery + proton, 39	90% ^a^	NR	Altered short-term memory, social and emotional functioning and significant school difficulties in children who had RT after several surgeries. Behavioral disorder rates lower after STR + RT.	NR
Confer *et al*. ^20^ (2012); N = 13	0.7	Surgery/biopsy + proton	85% ^a^	Grade 2 headache, 1	NR	0
Indelicato *et al*. ^14^ (2012); N = 40	0.7	Surgery/biopsy + proton therapy	100% ^a^	Emesis, 1; headache, 1; presyncope, 2; nausea, 9	None to date	0
Bishop *et al*. ^21^ (2014); N = 21	2.75	Surgery/biopsy + proton, 15; proton alone, 4	92%	NR	Vasculopathy, 2; endocrinopathy, 16	Secondary to surgically induced DI, 1
Merchant *et al*. ^22^ (2017); N = 94	2.65	Surgery/biopsy + proton therapy	97.8% (3-year)	NR	Preservation of academic achievement	NR

CVA, cerebrovascular accident; DI, diabetes insipidus; MCA, middle cerebral artery; NR, not reported; PD, progression of disease; RT, radiation therapy; STR, subtotal resection. ^**a**^Crude rate at the time of reporting.

## Spot scanning and intensity-modulated proton therapy

**Figure 2.**
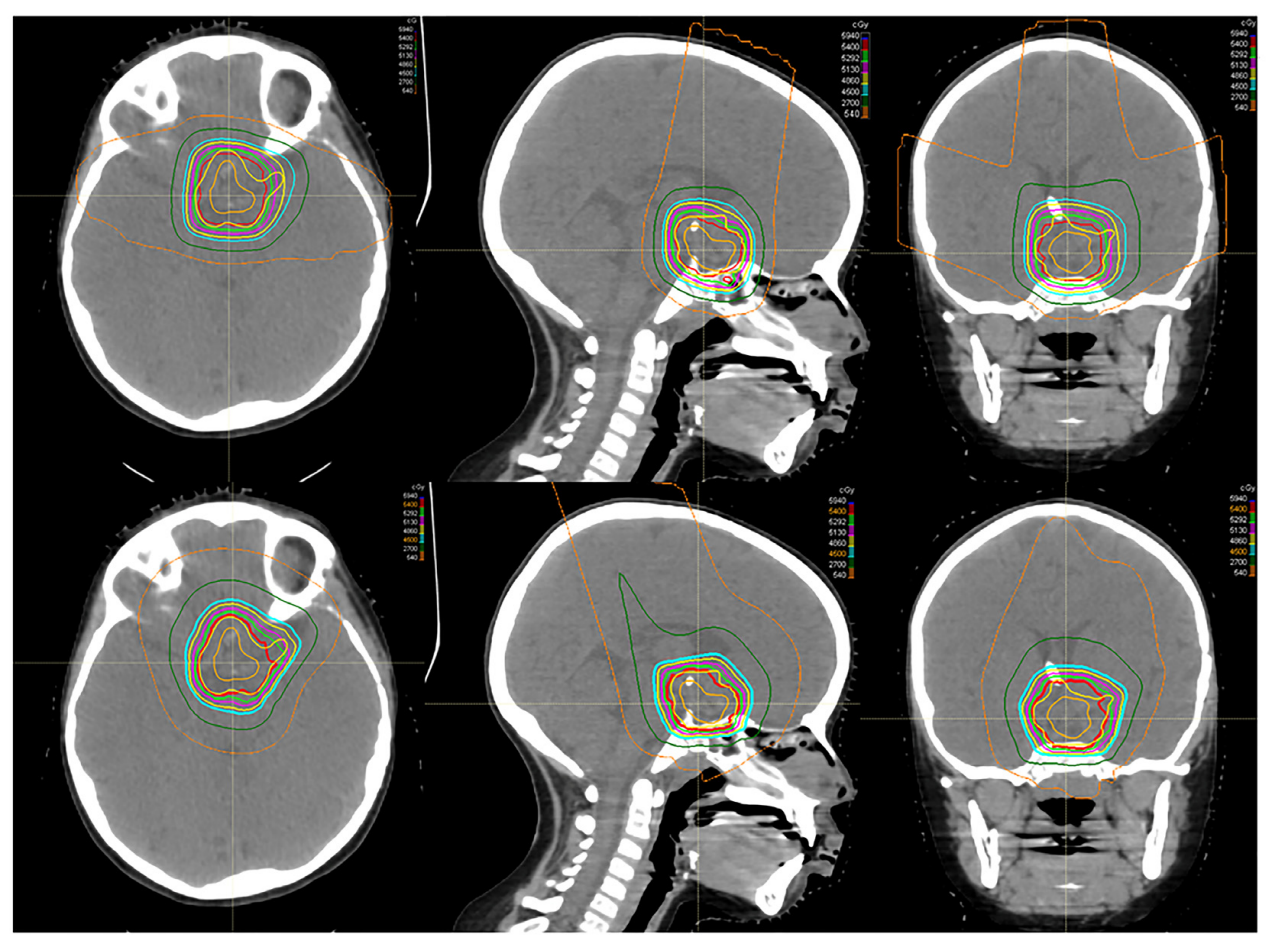
Dosimetric comparison of passive-scatter proton therapy and pencil-beam scanning. An example of the dose distribution for passive-scatter proton therapy (top) and pencil-beam scanning (bottom) in a patient with craniopharyngioma demonstrates comparable and acceptable target coverage in both plans. In this example, however, the pencil-beam modulation is used to create a more homogenous dose plan. The maximum doses to the optic chiasm are 55.6 Gy for the passive-scatter plan and 54.6 Gy for the pencil-beam scanning plan. The figures are original images taken in our clinic for this publication.

Spot scanning provides better proximal and distal target conformality compared with passive-scatter proton therapy by covering the target with small mono-energetic pencil beams steered by magnets. Dosimetric studies have shown that IMPT decreases the dose to normal surrounding tissue compared with double-scatter proton therapy ^23^, as shown in Figure 2. However, spot scanning is more sensitive to changes in the volume of cystic craniopharyngiomas during treatment, which could lead to underdosing at the margins. Furthermore, most spot-scanning systems do not allow aperture-based delivery, which means that the beam penumbra may be less conformal at the lateral target.

## Reducing radiation target margins

In radiation planning for all modalities, target volumes are delineated by using computed tomography simulation images fused to T1- and T2-weighted post-contrast thin-sliced (1 to 1.5 mm slice thickness) magnetic resonance imaging (MRI). The gross tumor volume (GTV) has historically been expanded by a margin of 10 mm to create the clinical target volume (CTV). However, in a prospective analysis of 88 children who received radiotherapy between 1998 and 2009, a 5 mm and a 10 mm expansion for the CTV provided comparable PFS ^1^. A recent phase II protocol (RT2CR, NCT01419067) at the University of Florida in conjunction with St Jude Children’s Research Hospital prospectively evaluated a 5 mm CTV margin using proton therapy. Early results are promising in terms of both disease control and reduced toxicity ^22, 24^.

## Adaptive planning

**Figure 3.**
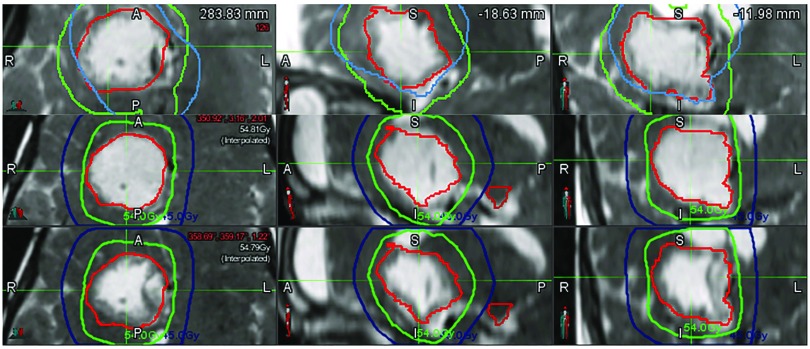
Dynamic cyst changes. Pictured are the T2-weighted magnetic resonance images (MRIs) in an axial, sagittal, and coronal presentation (left to right) taken weekly during radiotherapy for craniopharyngioma. The most superior MRI was obtained for radiation planning purposes, and the red line represents the contoured gross tumor volume. The images in the second row were obtained during the first week of radiation treatment and demonstrate cyst growth. The most inferior images were obtained during the second week of radiation treatment after the cyst had been drained via the Ommaya reservoir. The figures are original images taken in our clinic for this publication.

Improved conformality with the most recent technological advances in radiation therapy delivery increases the susceptibility of the proton dose distribution to the effects of dynamic cyst changes that occur throughout the 6 weeks of treatment. The GTV, by virtue of cyst reduction or enlargement, has been observed to change on average by 28.5% (range of −20.7% to 82%) during treatment when weekly MRIs are obtained ^10^. Weekly MRIs during radiotherapy are used to identify these changes, and adaptive planning is necessary when changes in tumor volume may impact target coverage, as shown in Figure 3. **

## Summary

Craniopharyngioma is a curable benign tumor treated primarily by conservative resection and radiotherapy. Reducing the late toxicities of radiotherapy remains of pivotal importance in treating craniopharyngioma. Recent technological advances in radiotherapy offer the promise of reducing side effects while maintaining high cure rates.

## Abbreviations

3DCPT, three-dimensional conformal proton therapy; 3DCRT, three-dimensional conformation radiation therapy; CTV, clinical tumor volume; EES, endonasal surgery; GTV, gross tumor volume; IMPT, intensity-modulated proton therapy; IMRT, intensity-modulated radiation therapy; MRI, magnetic resonance imaging; PFS, progression-free survival; STR, subtotal resection
